# Why the *C*-statistic is not informative to evaluate early warning scores and what metrics to use

**DOI:** 10.1186/s13054-015-0999-1

**Published:** 2015-08-13

**Authors:** Santiago Romero-Brufau, Jeanne M. Huddleston, Gabriel J. Escobar, Mark Liebow

**Affiliations:** Healthcare Systems Engineering Program, Mayo Clinic Robert D. and Patricia E. Kern Center for the Science of Health Care Delivery, 200 First Street SW, Rochester, MN 55905 USA; Division of Health Care Policy and Research, Department of Health Sciences Research, Mayo Clinic, 200 First Street SW, Rochester, MN 55905 USA; Division of Hospital Internal Medicine, Mayo Clinic, 200 First Street SW, Rochester, MN 55905 USA; Kaiser Permanente Division of Research, 2000 Broadway Avenue, 032 R01, Oakland, CA 94612 USA; Division of General Internal Medicine, Mayo Clinic College of Medicine, 200 First Street SW, Rochester, MN 55905 USA

## Abstract

Metrics typically used to report the performance of an early warning score (EWS), such as the area under the receiver operator characteristic curve or *C*-statistic, are not useful for pre-implementation analyses. Because physiological deterioration has an extremely low prevalence of 0.02 per patient-day, these metrics can be misleading. We discuss the statistical reasoning behind this statement and present a novel alternative metric more adequate to operationalize an EWS. We suggest that pre-implementation evaluation of EWSs should include at least two metrics: sensitivity; and either the positive predictive value, number needed to evaluate, or estimated rate of alerts. We also argue the importance of reporting each individual cutoff value.

## Introduction

Metrics typically used to report the performance of an early warning score (EWS), such as the area under the receiver operator characteristic curve (AUROC), *C*-statistic, likelihood ratio, or specificity, are not adequate for an operational evaluation in a clinical setting because they do not incorporate information about prevalence of the disease. These metrics are being used to drive decisions regarding which EWS to implement as part of the afferent limb of rapid response systems. The metrics have been used extensively in pre-implementation evaluations, both in peer-reviewed publications [1, 2] and in guidelines [3]. Some of these evaluations have led to the recommendation of the National Early Warning Score (NEWS) as the standard EWS in the British National Health System [1, 4], which is already being used in many hospitals [5–7].

These metrics are accepted and widely used to evaluate all types of diagnostic tools, and they are very useful in evaluating most other tools. The *C*-statistic, AUROC, specificity, or likelihood ratio are only dependent on the test and are not influenced by the pre-test probability (generally assumed to be equal to the prevalence). Using these metrics is generally useful for two reasons. First, pre-test probability may vary widely in the patients on which a physician decides to perform the diagnostic test. Since test assessors do not know which patients will undergo the test, it makes sense to leave that unknown out of the equation when evaluating the test. Second, the pre-test probability is usually in a clinically plausible range, and most clinical tests are performed on patients who may well have the disease or condition in question. EWSs are a special type of diagnostic tool, however, which makes these classic metrics not ideal. EWSs try to predict a condition whose prevalence is known to be less than 2 % [8, 9] in general care inpatients. As a result, these metrics (AUROC, *C*-statistic, specificity, likelihood ratio) provide incomplete information and can lead to overestimating the benefits of an EWS or underestimating the cost in terms of clinical resources.

## Prevalence is important

Performing diagnostic tests when the pre-test probability is low is accepted practice. We test for phenylketonuria in newborns and for HIV in blood samples from US blood donors, both conditions with an estimated pre-test probability of 0.0001 [10, 11].

Trying to predict events such as these with extremely low prevalence is justified when: 1) the event has severe consequences and the consequences increase if missed for a period of time; and 2) when the test administered is relatively easy and cheap to perform. Systems using EWSs to try to detect or predict physiological deterioration have these two characteristics. Causes of physiological deterioration typically have more severe consequences if treatment is delayed (e.g., sepsis and other causes of shock, respiratory insufficiency, etc.). In addition, the marginal cost of calculating a patient’s EWS is very small for paper-based EWSs, but even more so for automated EWSs embedded in the electronic medical record.

When the prevalence is very low, however, even “good” tests have surprisingly low post-test probability. The following example involved one of the authors of this article. Twenty years ago, an 18 year old donated blood and was told by letter that her HIV enzyme-linked immunosorbent assay (ELISA) test was repeatedly reactive while her western blot was indeterminate. When the author tried to determine the probability that she actually had HIV, he found that even though ELISA tests for HIV are extremely accurate, the pre-test probability (at that time) that an 18-year-old female college student was actually infected with HIV was 0.0002 [12]. Hence, because of the extremely low pre-test probability, even a positive result on a test with almost 100 % sensitivity and 99.5 % specificity [12–14] (which is equivalent to a positive likelihood ratio of 200) meant that, considering the test results, the probability that this woman was actually infected with HIV was only around 4 %.

## Most EWS operate in a low-prevalence environment

Why is prevalence so important? The positive predictive value (PPV) can be expressed as a function with a direct relationship to prevalence:

$$ \mathrm{P}\mathrm{P}\mathrm{V}=\frac{\mathrm{Sensitivity}\times \mathrm{prevalence}}{\mathrm{Sensitivity}\times \mathrm{prevalence}+\left[\left(1-\mathrm{specificity}\right)\times \left(1-\mathrm{prevalence}\right)\right]} $$

As prevalence decreases, so does the PPV. The post-test probability depends on the pre-test probability.

**Fig. 1:**
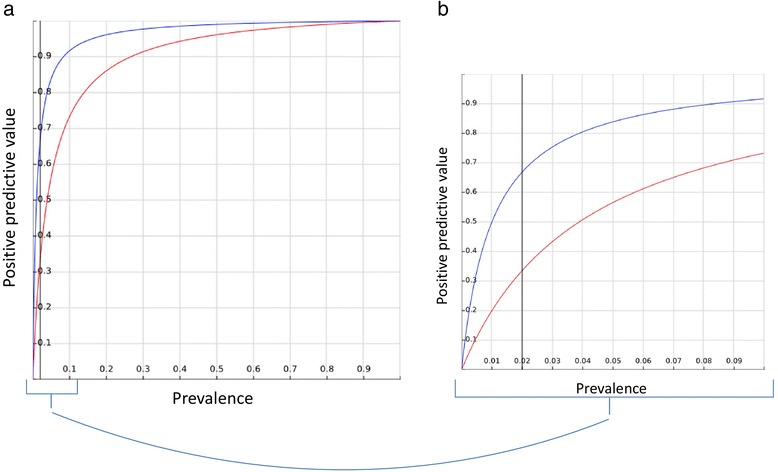
PPV as a function of prevalence for two sample scores (EWS): score A (blue), with a sensitivity of 99 % and a specificity of 99 %; and score B (red), with a sensitivity of 99 % and a specificity of 96 %. **a** Full range of possible PPV and prevalence, from 0 to 1. **b** Region of prevalence <0.1, adding a line to show an example prevalence of 0.02 (corresponding to an estimate of the rate of physiological deterioration of inpatients). A decrease of only 3 % in specificity can mean a 50 % decrease in PPV: from 0.33 to 0.66

Prevalence, here and in the rest of our article, refers to the pre-test probability, or the prevalence of the disease in the subset of patients in which the test is administered. Figure 1 demonstrates this function for a sensitivity of 99 % and specificities of 99 % and 96 %. Even with such extremely high sensitivity and specificity, it is easy to see how the PPV declines rapidly for pre-test probabilities <0.1. This means that for pre-test probabilities <0.1, a test with a high sensitivity and specificity may not necessarily produce a high post-test probability for a positive test.

In this setting, the sensitivity and specificity could inform what score and cutoff value performs better, but the magnitude of the difference could be very misleading. For example, for a sensitivity of 99 % and a prevalence of 0.02 outcomes per patient-day (as discussed previously, similar to the prevalence of physiological deterioration in inpatient populations in general care beds), reducing the specificity by only 3 % would halve the PPV to 33 % (see Fig. 1). The *C*-statistic or AUROC is perhaps the most commonly used metric in the EWS literature, especially in studies comparing different scores [15, 16]. The AUROC can be understood as the probability that a higher score distinguishes between two patients, one with the outcome and one without the outcome. Some of the limitations of using the AUROC for models predicting risk have been discussed previously [17]. We also previously showed how some scores with high AUROC did not perform well under simulation of clinical use using PPV as a metric [8]. In addition to lacking information about disease prevalence, the AUROC has the additional problem of summarizing information about different cutoff values, some of which will never be used because of unacceptably high false-positive rates. It is important to evaluate and report actionable cutoff values independently.

Accordingly, reliance only on metrics such as the *C*-statistic [15, 16] or the AUROC can offer misleading reassurance. We argue that in these settings it is better to report metrics which incorporate the pre-test probability.

## What metrics should we use?

If the commonly used metrics (sensitivity, specificity, likelihood ratio, and derived measures like the *C*-statistic or AUROC) do not seem to provide useful information to evaluate EWSs, what can be used instead? Patients who score above a threshold usually undergo further evaluation (a “workup”), so limiting false alerts is critical to avoid alarm fatigue [18, 19] and overuse of clinical resources.

Ideally, reports on the performance of EWSs would include information about both goals of the EWS: detecting a high percentage of outcomes, and issuing few false-positive alerts. This makes a tradeoff evident: the benefit of the system is the early detection, and the main burden or cost is the false-positive alerts. To evaluate the first aim (the benefit), sensitivity can be a good metric because it provides the percentage of outcomes that the score is able to predict within a specified timeframe. To evaluate the second aim (the clinical burden), there are a few metrics that can be used. These metrics include the PPV, the number needed to evaluate (NNE), also known as the workup to detection (WTD) ratio, and the estimated rate of alerts.

**Fig. 2:**
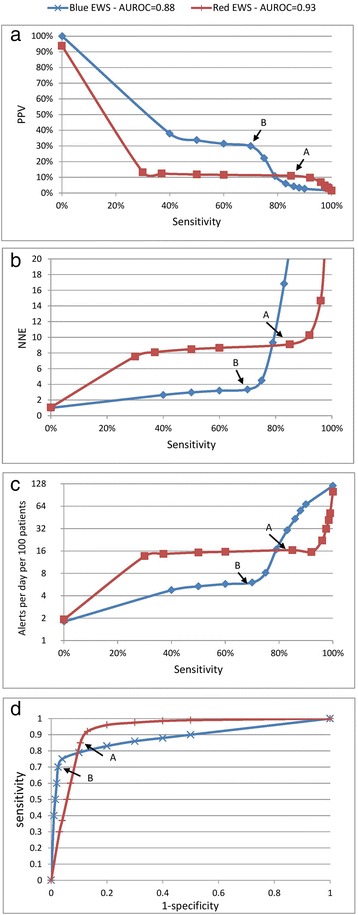
Graphic representations of the proposed metrics and the ROC curves for all cutoff values of two sample scores (EWS). **a**, **b**, **c** Three proposed metrics for the two sample scores. **d** ROC curves which we suggest not using. Each point in the graphs corresponds to a threshold of a specific score. Points A and B are referred to in the text. *AUROC* area under the receiver operator characteristics, *EWS* early warning score, *NNE* number needed to evaluate, *PPV* positive predictive value

The PPV would provide the percentage of alerts that are followed by an outcome within a certain number of hours. This tells us the percentage of alerts which are useful in that they precede an outcome. To use the PPV effectively we need to overcome some preconceptions about what is a good PPV. In a classic diagnostic tool, a PPV lower than 50 % is generally unacceptable, because this would mean that one-half of the people with a positive test result would be incorrectly classified as having the condition. In an EWS, however, this only means having to perform further workup on two patients for each outcome correctly predicted. This approach may be acceptable to predict severe outcomes that can often result in death, and only requires a brief assessment to confirm or discard the “diagnosis” of physiological deterioration (Fig. 2a).

The NNE (using parallelism with the number needed to treat) is the number of patients that it is necessary to further evaluate (or workup) to detect one outcome. It is a direct measure of the cost-efficiency of each alert. A PPV of 20 % is equivalent to an NNE of 5 (Fig. 2b).

The estimated rate of alerts provides the estimated number of alerts (workups needed) per unit of time per number of inpatients monitored. For example, one can estimate the number of alerts per day per 100 inpatients. This can guide discussions with practicing providers. Once an EWS is in place, the number of alerts can be “titrated” by changing the alert threshold. Based on our experience, there seems to be a “sweet spot” in the number of daily alerts: too many will create alarm fatigue, but too few can lead to unfamiliarity with the clinical response workflow (Fig. 2c).

## Graphic representation

Figure 2 uses two sample scores (Red EWS and Blue EWS) to exemplify use of the recommended principles and metrics, and to illustrate some of the recommendations and warnings. As can be seen in Fig. 2d, the Red EWS has a higher AUROC of 0.93, as compared with an AUROC of 0.88 for the Blue EWS, which might lead to choosing the Red EWS over the Blue EWS. However, we can see that the other metrics tell a different story, and offer more important information about the consequences of using the scores. If we look at Fig. 2a, we can see that the maximum PPV of the Red EWS is only about 11 % at a sensitivity of 85 % (Point A), so a clinician would need to respond to 16 calls per day for every 100 patients (Fig. 2c) and for every nine patients evaluated only one would be a true positive (NNE = 9; see Fig. 2b). This is likely to disrupt the clinical workflow significantly and create alarm fatigue. The Blue EWS, on the other hand, for a slightly lower sensitivity of 70 %, has a PPV of 30 % (Point B). This would create six alerts per day for every 100 patients, and one in every three alerts would be a true positive (NNE = 3; see Fig. 2b). So when comparing Points A and B, a tradeoff is evident: Point A (Red EWS) has a sensitivity 21 % higher (85 % vs. 70 %), but the rate of alerts is 166 % higher (16 per day vs. 6 per day). The Blue EWS offers a more manageable and useful prediction. In general, if the receiver operating characteristic (ROC) curves for two different EWSs intersect, even the score with the lowest AUROC may perform better in certain circumstances.

## Additional considerations

In addition to what metrics to use, there are some additional aspects to be considered when evaluating a specific EWS.

The likelihood ratio can also be considered for the evaluation of an EWS. Likelihood ratios are the multiplier that needs to be applied to the pre-test odds to calculate the post-test odds (the positive or negative likelihood ratio in the case of a positive or a negative result in the test, respectively). These ratios are one step closer to providing a clear cost–benefit analysis, because they only need to be multiplied by a prevalence or event rate to provide an estimation of cost in terms of false alerts. However, they still do not make the tradeoff evident.

Metrics that focus on missed events, such as the negative predictive value, are mainly useful if the intended use of the EWS is to rule out the possibility of physiological deterioration. This does not seem to be the current intended use, which, rather, is to add “an additional layer of early detection” [4, 20].

Reclassification indices can also be considered. These indices can offer good comparisons between two different scores, by showing how many additional patients would be correctly classified as having an event or not when one score is used over another. However, reclassification indices are limited in that they are only able to compare scores one-to-one, and they provide only comparisons, not results in absolute terms: a score may correctly classify double the number of patients, but this does not mean the resulting PPV will be actionable. Reclassification indices do not allow for direct evaluation of the tradeoff between detection and false alerts in absolute terms.

Just as the measures used to evaluate a diagnostic test (e.g., to measure the accuracy of a specific HIV diagnostic test) are different from the evaluation of the strategy (answering the question “does testing blood for HIV reduce infections?”), the pre-implementation metrics discussed in this paper (aimed at evaluating the accuracy of the EWS) are different from post-implementation “success measures” of the strategy (aimed at answering the question “does the use of EWS improve patient outcomes?”).

EWSs are really trying to predict instances of physiological deterioration. Surrogate measures of physiological deterioration include ICU transfers and cardiorespiratory arrests, and some authors also include the calls to the rapid response team. These proxy outcomes vary locally by hospital and patient population, but they are within the same order of magnitude (0.02) so the arguments made in this article still hold true despite those variances. We nonetheless recommend reporting the prevalence of physiological deterioration in studies comparing EWSs.

Our article assumes selection of a threshold to trigger an escalation of care. Threshold selection has been described as a function of the test’s properties (sensitivity and specificity), the prevalence of the condition, and the benefit or harm of identifying or missing the diagnosis of a condition [21]. Different hospitals may have different priorities or constraints that may affect any of these variables, but we believe the metrics should make evident the tradeoff between detection of physiological deterioration and the practice constraints.

## Final remarks

We have discussed the limitations of metrics that do not incorporate information regarding prevalence. In broader terms, we can divide metrics into two groups. First, a group that focuses on the ranking of scores that does not take clinical utility into consideration, using metrics widely used in statistical science to evaluate other types of classification systems (e.g., systems used in credit card fraud [22] or the prognosis of alcoholic hepatitis [23]). Second, another group that is specific to the problem of operationalizing EWS and tries to predict the operational consequences related to using one score over another. In the first group we find the aforementioned metrics (sensitivity, specificity, likelihood ratios, or AUROC), while in the second group we find metrics such as the PPV or the NNE.

To compare EWSs it is important to report metrics that incorporate the extremely low prevalence. We recommend using the PPV, the NNE and/or the estimated rate of alerts combined with sensitivity to evaluate each of the plausible score cutoff values. Including two of these metrics in a graph allows for easy evaluation of practical clinical usefulness both in absolute terms and for comparison of two or more EWSs. Evaluating EWSs in this way demonstrates the balance between the benefit of detecting and treating very sick patients with the associated clinical burden on providers and patients.

Clinically, EWSs should not replace clinical judgment and decision-making but should serve as a safety net.

## Abbreviations

AUROC: Area under the receiver operating characteristic
ELISA: Enzyme-linked immunosorbent assay
EWS: Early warning score
NEWS: National Early Warning Score
NNE: Number needed to evaluate
PPV: Positive predictive value
ROC: Receiver operating characteristic
WTD: Workup to detection

## References

[1] Smith GB, Prytherch DR, Meredith P, Schmidt PE, Featherstone PI (2013). The ability of the National Early Warning Score (NEWS) to discriminate patients at risk of early cardiac arrest, unanticipated intensive care unit admission, and death. Resuscitation.

[2] Tirkkonen J, Olkkola KT, Huhtala H, Tenhunen J, Hoppu S (2014). Medical emergency team activation: performance of conventional dichotomised criteria versus national early warning score. Acta Anaesth Scand.

[3] Acutely ill patients in hospital: recognition of and response to acute illness in adults in hospital. National Institute for Health and Care Excellence. 2007. www.nice.org.uk/guidance/cg50. Accessed 02 Jul 2015. 21204323

[4] National Early Warning Score (NEWS): standardising the assessment of acute-illness severity in the NHS—report of a working party. Royal College of Physicians of London. 2012. www.rcplondon.ac.uk/resources/national-early-warning-score-news. Accessed 02 Jul 2015.

[5] D’Cruz R, Rubulotta F (2014). Implementation of the National Early Warning Score in a teaching hospital [Abstract 0567]. Intensive Care Med.

[6] Gleeson L, Reynolds O, O’Connor P, Byrne D (2014). Attitudes of doctors and nurses to the National Early Warning Score System. Irish J Med Sci.

[7] Jones M (2012). NEWSDIG: The National Early Warning Score Development and Implementation Group. Clin Med.

[8] Romero-Brufau S, Huddleston JM, Naessens JM, Johnson MG, Hickman J, Morlan BW, Jensen JB, Caples SM, Elmer JL, Schmidt JA (2014). Widely used track and trigger scores: are they ready for automation in practice?. Resuscitation.

[9] Escobar GJ, LaGuardia JC, Turk BJ, Ragins A, Kipnis P, Draper D (2012). Early detection of impending physiologic deterioration among patients who are not in intensive care: development of predictive models using data from an automated electronic medical record. J Hosp Med.

[10] Donlon J, Levy H, Scriver C, Scriver CEA (2004). Hyperphenylalaninemia: phenylalanine hydroxylase deficiency. The metabolic and molecular bases of inherited disease.

[11] Dodd RY, Notari EP, Stramer SL (2002). Current prevalence and incidence of infectious disease markers and estimated window-period risk in the American Red Cross blood donor population. Transfusion.

[12] Burkhardt U, Mertens T, Eggers HJ (1987). Comparison of two commercially available anti-HIV ELISAs: Abbott HTLV III EIA and Du Pont HTLV III-ELISA. J Med Virol.

[13] Stetler HC, Granade TC, Nunez CA, Meza R, Terrell S, Amador L, George JR (1997). Field evaluation of rapid HIV serologic tests for screening and confirming HIV-1 infection in Honduras. Aids.

[14] McAlpine L, Gandhi J, Parry JV, Mortimer PP (1994). Thirteen current anti-HIV-1/HIV-2 enzyme immunoassays: how accurate are they?. J Med Virol.

[15] Smith GB, Prytherch DR, Schmidt PE, Featherstone PI (2008). Review and performance evaluation of aggregate weighted “track and trigger” systems. Resuscitation.

[16] Smith GB, Prytherch DR, Schmidt PE, Featherstone PI, Higgins B (2008). A review, and performance evaluation, of single-parameter “track and trigger” systems. Resuscitation.

[17] Cook NR (2007). Use and misuse of the receiver operating characteristic curve in risk prediction. Circulation.

[18] Graham KC, Cvach M (2010). Monitor alarm fatigue: standardizing use of physiological monitors and decreasing nuisance alarms. Am J Crit Care.

[19] Hannibal GB (2011). Monitor alarms and alarm fatigue. AACN Adv Crit Care.

[20] Early warning systems: scorecards that save lives. Institute for Healthcare Improvement. http://www.ihi.org/resources/Pages/ImprovementStories/EarlyWarningSystemsScorecardsThatSaveLives.aspx. Accessed 02 Jul 2015.

[21] Jund J, Rabilloud M, Wallon M, Ecochard R (2005). Methods to estimate the optimal threshold for normally or log-normally distributed biological tests. Med Decis Making.

[22] Hand DJ, Whitrow C, Adams NM, Juszczak P, Weston D (2008). Performance criteria for plastic card fraud detection tools. J Oper Res Soc.

[23] Srikureja W, Kyulo NL, Runyon BA, Hu KQ (2005). MELD score is a better prognostic model than Child–Turcotte–Pugh score or discriminant function score in patients with alcoholic hepatitis. J Hepatol.

